# Image‐guided bolus electron conformal therapy – a case study

**DOI:** 10.1120/jacmp.v12i1.3311

**Published:** 2010-10-07

**Authors:** Omar A. Zeidan, Bhavin D. Chauhan, William W. Estabrook, Twyla R. Willoughby, Rafael R. Manon, Sanford L. Meeks

**Affiliations:** ^1^ Department of Radiation Oncology M.D. Anderson Cancer Center Orlando Orlando FL 32806 USA

**Keywords:** image‐guided radiotherapy, bolus electron conformal therapy

## Abstract

We report on our initial experience with daily image guidance for the treatment of a patient with a basal cell carcinoma of the nasal dorsum using bolus electron conformal therapy. We describe our approach to daily alignment using treatment machine‐integrated megavoltage (MV) planar imaging in conjunction with cone beam CT (CBCT) volumetric imaging to ensure the best possible setup reproducibility. Based on MV imaging, beam aperture misalignment with the intended treatment region was as large as 0.5 cm in the coronal plane. Four of the five fractions analyzed show induced shifts when compared to digitally reconstructed radiographs (DRR), in the range of 0.2−0.5 cm. Daily inspection of CBCT images show that the bolus device can have significant tilt in any given direction by as much as 13° with respect to beam axis. In addition, we show that CBCT images reveal air gaps between bolus and skin that vary from day to day, and can potentially degrade surface dose coverage. Retrospective dose calculation on CBCT image sets shows that when daily shifts based on MV imaging are not corrected, geometrical miss of the planning target volume (PTV) can cause an underdosing as large as 14% based on DVH analysis of the dose to the 90% of the PTV volume.

PACS number: 87.55.kh

## I. INTRODUCTION

Bolus electron conformal therapy (ECT) uses a single electron beam with a variable thickness bolus or compensator to treat superficial target volumes. Bolus ECT is particularly useful for the treatment of shallow tumors where conventional photon treatments may not be desirable due to lower surface doses and higher exit doses. The bolus is patient‐specific and is designed for shaping the distal 90% dose surface to conform and contain the planning target volume (PTV) while delivering minimal dose to adjacent, underlying critical structures and normal tissues. The delivery of ECT can be in principle achieved through a variety of approaches such as the use of a retractable electron multileaf collimator (eMLC),^(^ ^1^ ^)^ modulation of energy and intensity of electron beams^(^ ^2^ ^)^ and, most recently, use of existing photon‐based MLCs for electron fluence modulation.^(^ ^3^ ^)^ However, these approaches are far from being mainstream for ECT delivery due to the fact that they exist primarily as prototypes in academic institutions or because commercially available treatment planning systems (TPS) do not support them.

Currently, the most clinically established approach to ECT is the use of solid machinable bolus devices that are placed directly on the patient's skin. Several clinical studies have shown the clinical efficacy of this technique for postmastectomy irradiations,^(^ ^4^ ^)^ head and neck (H&N),^(^ ^5^ ^)^ and paraspinal muscle treatment.^(^ ^6^ ^)^ The common clinical implementation of bolus ECT, as described in the previous studies, is to perform a visual inspection of the bolus on the patient using planning CT images. This QA process is performed once before the start of treatment. None of the previous studies have attempted to characterize the performance of the bolus during the course of treatment using image‐guidance techniques or otherwise. Subsequently, there is no available information from these studies on the quality of coverage to the PTV or on the sparing of sensitive structures due to daily setup uncertainties or daily variations in bolus placement.

Our institutional experience with image guidance shows the presence of interfraction motion in most patients leading to non‐trivial setup uncertainties even in the least motion‐prone regions such as in the H&N.^(^ ^7^ ^,^ ^8^ ^)^ As with any highly conformal photon‐based radiation therapy, image guidance is becoming the mainstream approach for daily alignment and localization. Bolus ECT is a highly‐conformal therapy and, as such, image guidance should be a crucial component of this treatment process. Therefore, we explore in this case study the use of daily image guidance to evaluate the performance of bolus ECT and quantify dosimetric effect due to setup uncertainties. Daily image guidance was achieved using two independent imaging modalities integrated within the treatment machine: volumetric imaging using CBCT and planar MV portal imaging using an electronic portal imaging device (EPID). Imaging data were later used to assess retrospectively the dosimetric effect of setup uncertainties, and for the evaluation of the quality of bolus fit and orientation to patient skin.

## II. MATERIALS AND METHODS

### A. Planning and QA with bolus ECT

The case study presented here is of a patient with localized skin lesion of the right nasal dorsum with the intention of definitive radiotherapy. Due to the location of the lesion and nearby critical structures, the clinical assessment mandated the use of the bolus ECT for treatment. The design and QA process of the bolus requires two independent CT scans of the patient. The first CT scan is a preplan scan in which the patient is scanned in the supine position with a Timo headrest (CIVCO Medical Solutions, Orange City, IA) and a thermoplastic mask with three external BBs for localization. The use of the mask is strictly for setup reproducibility and it is removed before the CT scan is acquired. A crosshair is drawn using a permanent marker on the bolus surface facing the beam with help of localization lasers inside the CT room. This mark is then used to line up the bolus to the crosshair mark of the light field inside the treatment room to insure proper alignment of the beam axis with the bolus. After the CT study is acquired, the data are exported to the ${\text{Pinnacle}}^{3}$ (Philips Medical Systems, Fitchburg, WI) treatment planning system (TPS) where skin, critical structures and PTV contours were drawn. The PTV volume was $10.1\;{\text{cm}}^{3}$ with an approximate areal size of nearly 2.5×4 cm from a beam's eye view (BEV) in the AP direction and extended to a maximum depth of nearly 3 cm from skin. The PTV volume was determined by the physician based on clinical examination and CT images rather than on GTV or CTV expansions. A cone size of 6 cm× 6 cm was determined to be adequate to encompass the PTV at a source to skin distance (SSD) of 105 cm to the bolus surface using an AP beam. Prescription (Rx) dose to target volume was 200 cGy per fraction using a 9 MeV electron beam. The dose prescription was selected such that the distal 90% dose surface conforms to the PTV volume while delivering minimal dose to the adjacent lens of the right eye.

The bolus design process starts by exporting the DICOM RT information into the p.d. software (version 4.0, .decimal, Inc., Sanford, FL). Using the RT structure information and beam parameters, the software optimizes the bolus shape to meet the Rx and coverage requirements. No dose information is available at this stage. A careful 3D visual inspection is performed in the p.d. software to determine the quality of fit on patient skin. Further modification of the bolus within the software may be necessary. Once a satisfactory bolus design is determined, the bolus information is sent to the TPS and dose is calculated. The radiation oncologist examines the plan and determines its appropriateness based on PTV coverage and dose homogeneity. If no further changes to the bolus are needed, the coordinates of the proximal and distal bolus surfaces are exported from the p.d software directly to the manufacturer (.decimal, Inc.) for precision bolus fabrication. The bolus ECT device is made from hard machinable wax (Machinable Wax, Inc., Lake Ann, MI) material with radiation properties previously described by Low and Hogstrom.^(^ ^9^ ^)^ The electron bolus design and algorithms were developed by Low et al.^(^ ^10^ ^)^ Once the bolus is shipped from the manufacturer, a second CT of the patient is performed with the same setup conditions as the preplan scan but with the fabricated bolus in place. This planning CT set is exported to the TPS for a final dose calculation and a final examination of the quality of fit of the bolus on the patient. The radiation oncologist inspects the target dose coverage from the final calculated plan to insure similarities between this plan and the original prebolus fabrication plan.

### B. Treatment setup and daily imaging

**Figure 1:**
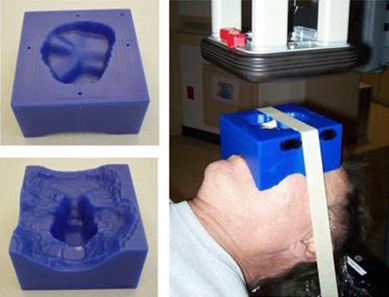
Pictures of the bolus electron compensator (left) and patient setup (right). The bolus surface facing the electron beam is shown in the top left picture.

**Figure 2:**
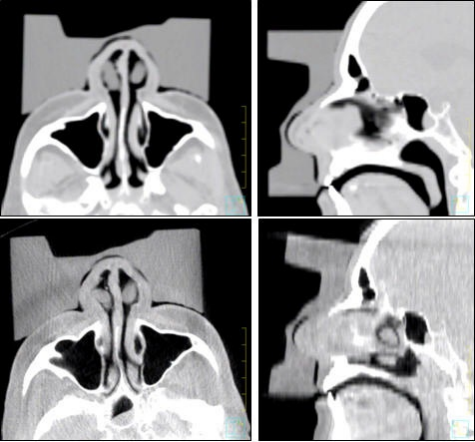
Axial and sagittal images of the reference plan CT (top) and a set of CBCT images from one of the treatment fractions (bottom). Notice the magnitude of tilt of the bolus in both the axial and sagittal CBCT images with respect to the planning images.

The patient is localized daily to room lasers using mask fiducials. The mask is then removed carefully to minimize potential changes of the patient head position within the Timo. The bolus is then placed on the patient and secured in position using a masking tape that attaches the bolus to the treatment couch. Figure 1 shows a picture of the bolus used in our study and the patient setup. The therapist use a level placed on the bolus flat edges that face the beam, as seen in the top left picture of Fig. 1, to ensure bolus levelness in the coronal plane. Volumetric CBCT images are then acquired using the Varian on‐board imager (OBI) that is integrated within the treatment linac (Varian 23iX, Varian Medical Systems, Palo Alto, CA). The CBCT images are inspected at the OBI console by therapists and a physicist in the axial and sagittal planes to ensure a snug fit of the bolus device to patient skin and to determine if there is any noticeable tilt of the bolus device in either plane. Occasional re‐adjustment of the bolus to minimize either noticeably large air gaps (>0.5 cm) or to level the bolus device due to tilt are needed post‐CBCT imaging. Once the placement of the bolus is determined to be adequate, the electron cone and aperture are attached to the linac head. Figure 2 (top) shows axial and sagittal images of the bolus ECT used for planning. The images in Fig. 2 (bottom) show an example of axial and sagittal views from a daily CBCT image set.

**Figure 3:**
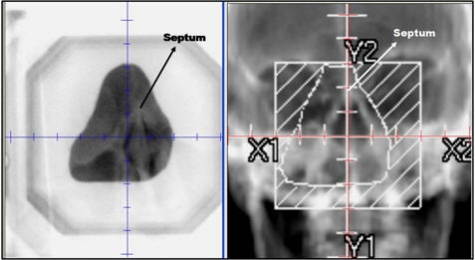
MV port image (left) of electron cone and aperture. The corresponding DRR (right) shows the intended treatment region based on bony anatomy.

For the last step of the setup verification process, an MV image is acquired with the electron cone aperture in place, as shown in Fig. 1. The planar MV image is examined by the therapist at the OBI console and compared side‐by‐side to beam DRR (digitally reconstructed radiograph) generated by the TPS. In this case study, the nasal septum bony structure was used as the alignment surrogate for daily shifts in the superior‐inferior and lateral directions (i.e., coronal plane). Figure 3 (left) displays an MV image of the electron port showing major bony anatomy structures underlying the treatment region and the intended treatment region (Fig. 3 (right)).

### C. Dose recalculation

In order to estimate the dosimetric effect on PTV coverage from daily image‐induced shifts, daily CBCT images were imported from the OBI software to the TPS for dose recalculations. A total of five randomly selected fractions were chosen over five treatment weeks (one fraction per week) for this retrospective dose analysis. Image fusion of each CBCT image set to the planning CT image set was performed in the Syntegra module of the Pinnacle TPS. This allows for the transfer of RT structures from the planning image set to the CBCT image sets. Before dose calculation is performed on the CBCT images, the shifts from each alignment are translated to the beam position in the TPS to reflect the actual beam position with respect to the bolus. This simulates the effects of misalignment on PTV dose coverage had the shifts not been applied on the patient. It is worth mentioning that our examination of daily CBCT images over the course of treatment showed no detectable changes in the patient's nose morphology that could potentially affect PTV coverage. However, this may not be the case in other anatomical locations and, therefore, CBCT can be used as an important tool to detect significant changes in patient anatomy that may warrant replanning with a new or modified bolus to accommodate these changes.

Dose calculations on CBCT images have been investigated by several studies and compared to dose calculation on image sets from conventional CT scanners. These studies show that CBCT images currently do not meet the performance specifications of conventional diagnostic‐quality CT scanners. This is particularly true in key imaging properties such as CT number accuracy, stability and linearity.^(^ ^11^ ^,^ ^12^ ^)^ In addition, the dosimetric differences between dose calculations in inhomogenous tissue regions between CT‐based treatment planning and CBCT‐based treatment planning were shown to be significant.^(^ ^13^ ^)^ Hence, due to the above factors, dose calculations on CBCT image sets in our study will be inaccurate – especially due to the presence of the nasal cavity structures in the electron beam path. Therefore, uniform density override of patient tissue and bolus material is necessary to perform dose calculation on the CBCT image sets. The patient tissue was uniformly assigned a density of $1.0\;\text{gm}/{\text{cm}}^{3}$ while the density of the bolus material was assigned the actual density of the bolus material of $0.92\;\text{gm}/{\text{cm}}^{3}$. Similarly, the same density overrides were performed on the reference CT image set to allow for accurate relative dose comparison between planned and daily delivered dose distributions. Dose volume histograms were generated from each fraction calculation and exported at 1.0 cGy bin resolution for comparison to the reference CT plan DVH. We used the dose corresponding to the 90% of the PTV volume (D90) and the percent PTV volume corresponding to the 200 cGy (Rx dose) or V200 as our quantifying metrics.

## III. RESULTS & DISCUSSION

A compilation of the absolute shifts that the therapist performed for each fraction based on daily MV imaging is summarized in Table 1. The maximum observed shift in any direction was 0.5 cm as seen from Fig. 3. A shift in one or more direction was performed in four of the five fractions analyzed (80%). These shifts are normally not obvious based on visual alignment of the bolus inside the treatment room. Therefore, our results indicate that traditional alignment of the bolus with beam axis based on light field alone may be inadequate for bolus ECT treatments.

**Table 1:** Magnitudes of daily setup shifts in the coronal plane based on EPID imaging for the left/right (L/R) and superior/inferior (S/I) directions.

*Fraction*	*L/R shift (cm)*	*S/I shift (cm)*
1	0.5	0.5
2	0.0	0.0
3	0.5	0.0
4	0.3	0.0
5	0.2	0.3

Visual analysis of CBCT daily image sets provides two important pieces of information. First, CBCT images reveal the quality of bolus conformality to patient skin by performing slice‐by‐slice inspections while evaluating air gap pockets between skin and bolus surfaces. We found that the air gaps based on daily CBCT image inspection, in comparison to the reference CT, vary slightly from day to day. The average and standard deviations of the gaps from all fractions were measured in the vicinity of the PTV and correspond to the anterior‐posterior gap (i.e., parallel to beam). The maximum observed gap was 5 mm in one fraction with an average and standard deviation of 2.8 and 1.0 mm, respectively. This is comparable to the maximum observed gap of 3.0 mm measured in the reference kVCT with bolus. Second, CBCT images can reveal potential bolus tilts with respect to beam axis at the time of treatment. Bolus device tilt can be present in any direction (clockwise or counterclockwise) when viewed in the sagittal and axial planes. For one the fractions, the measured tilt was as large as 7° and 13° with respect to beam axis in the axial and sagittal planes, respectively. The magnitude of tilt was determined retrospectively on the image display monitor as shown in Fig. 2. The presence of tilts in our case study may be potentially caused by either improper initial placement of the bolus device, patient intrafraction variations of neck roll and angulations, or both. These tilts may change the effective path of travel of the electron in the bolus and subsequently may compromise the coverage of the PTV and potentially induce undesirable air gaps that cause underdosing of the skin surface. Therefore, for similar cases to one discussed in this study the use of a Timo headrest and a thermoplastic mask for patient daily setup is beneficial in reducing tilts. During the course of treatment, a total of four adjustments (13% of the time) to the bolus device were performed. Those adjustments were triggered based on therapist evaluation of the CBCT images when large air gaps compared to the usual were observed or if significant tilts were observed after CBCT similar to the magnitude of tilts shown in Fig. 2. Following each bolus device adjustment, a second CBCT was performed to ensure device repositioning yielded better overall fit compared to the initial setup. It is worth noting that the determination of the quality of fit is largely a qualitative judgment by the therapist at this point, and we have not yet established an action level for adjustment in our clinic. Retrospective review by the physician of CBCT images for fractions with device adjustments shows an overall agreement with the therapist on the need for adjustment for those fractions.

**Figure 4:**
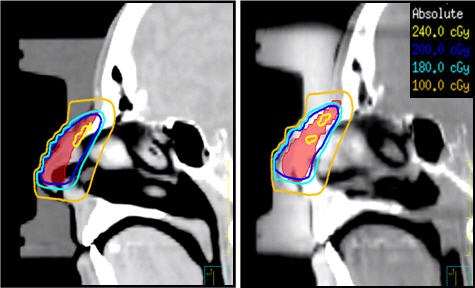
A sagittal dose distributions for the reference CT data (left) and of a CBCT dataset (right). The PTV is shown as a wash contour. The isodose lines shown correspond to the 120%, 100%, 90% (Rx line), and 50% isodose lines.

**Figure 5:**
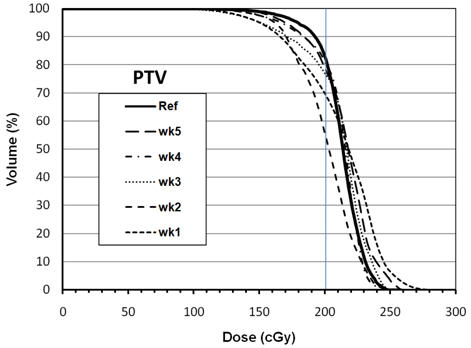
DVH overlays of PTV for all five fractions with the reference plan DVH for comparison (solid line). The vertical line at 200 cGy is shown to help estimate the degree of variation of V200 between all fractions. Shift corrections based on daily imaging has not been applied to these fractions.

The combined effect of misalignment and tilt of the bolus device on PTV coverage can be estimated by dose recalculation on CBCT image sets. Figure 4 shows an example of dose calculation in the sagittal plane on the reference image set (left) and the CBCT image set (right). Isodose distributions of the 120%, 100% (200 cGy), 90% (Rx line) and 100 cGy are shown. The PTV is shown as wash contour. Isodose coverage is very similar in both cases, and the depth of the 90% isodose line from skin in each case is very similar to within a millimeter (measurement error) at all depths within the treatment region. Therefore, we can safely assume that CBCT and CT images with similar bolus positioning and with density overrides will yield very similar dose distributions. Hence, the effect of misalignment and tilt from all five fractions should represent an actual dosimetric difference between each fraction calculation and the benchmark dose information from planning data. Figure 5 shows an overlay of DVHs of the PTV structure from all fractions. The planning DVH is shown as a thick solid line. The graph shows the magnitude of variation of PTV coverage due to daily misalignments when no shift correction is applied through image guidance. Regardless of the magnitude and direction of shift of each fraction, the PTV coverage will be compromised if the shifts are not applied to everyday treatment. In addition, we observed hot spots in the PTV volume as large as 300 cGy, as reflected by the DVH tails in Fig. 5. The quality of coverage can be estimated by the D90 value for each fraction. Table 2 summarizes the D90 per shift. The largest observed deviation occurred for week 1 fraction with a D90 value that is 14% less than the D90 for the reference plan. There is no clear correlation between the magnitude of shift shown in Table 1 for a given fraction and the corresponding D90 for that fraction. This could potentially be due to the dosimetric interplay effects of bolus tilt and misalignment. The volume of PTV covered by the 200 cGy Rx dose shows a larger magnitude of variations than the D90, as shown in Table 2. The most significant deviation of V200 is for week 2 fraction, with a difference of 35% less in PTV volume coverage compared to reference plan V200. Another important aspect of dose delivery variation due to bolus misalignment and tilt is the dose delivery variation in nearby sensitive structures to the PTV such as the right lens. The deviation in lens dose per fraction is indicated in the last column of Table 2. The maximum observed dose deviation from the reference plan is 11.4 cGy in fraction one. However, lens dose deviations seem to randomly vary between negative and positive values for the five fractions analyzed in this work and, therefore, may potentially cancel out any over‐ or underdosage effects in the lens by the end of the treatment course.

**Table 2:** Summary of D90 and V200 values of the PTV volume per fraction.

*Fraction #*	*D90 (cGy)*	*V200 (%)*	*Lens Dose Deviation (cGy)*
1	165	70	+11.4
2	171	54	+9.1
3	170	76	−3.7
4	183	80	−1.9
5	185	79	+7.5
Ref Plan	192	83	0

## IV. CONCLUSIONS

This case study explores the advantage of using daily imaging in conjunction with bolus ECT for the treatment of nasal lesions. By using two imaging modalities integrated with the treatment machine, daily MV planar and volumetric CBCT image were acquired and retrospectively analyzed for five randomly selected fractions. Planar MV imaging reveals potential misalignments of the beam axis with respect to the patient and bolus device in the coronal plane when compared to beam DRR. In our case study, this occurred in four of the five fractions analyzed. Volumetric CBCT images reveal the quality of fit of the bolus on patient skin and any potential bolus device tilt in the sagittal or axial planes. This quantitative information on daily setup variations are normally unavailable based on traditional visual setup procedures currently being performed for this type of treatment. Retrospective dose calculation on randomly selected CBCT fractions reveal that dose deviations as large as 14% can be present if daily shifts are ignored based on D90 analysis of the PTV. Geometrical misses of the PTV due to beam misalignments as revealed by MV images were as large as 0.5 cm in the coronal plane. Based on our findings, we recommend that daily image guidance be an integral component in bolus ECT treatments to ensure optimal dose coverage of PTV and dose sparing of critical structures.

## References

[1] Hogstrom KR , Boyd RA , Antolak JA , Svatos MM , Faddegon BA , Rosenman JG . Dosimetry of a prototype retractable eMLC for fixed‐beam electron therapy. Med Phys. 2004;31(3):443–62. 15070241 10.1118/1.1644516

[2] Ma CM , Pawlicki T , Lee MC , et al. Energy‐ and intensity‐modulated electron beams for radiotherapy. Phys Med Biol. 2000;45(8):2293–311. 10958195 10.1088/0031-9155/45/8/316

[3] Klein EE , Mamalui‐Hunter M , Low DA . Delivery of modulated electron beams with conventional photon multileaf collimators. Phys Med Biol. 2009;54(2):327–39. 19098355 10.1088/0031-9155/54/2/010

[4] Perkins GH , McNeese MD , Antolak JA , Buchholz TA , Strom EA , Hogstrom KR . A custom three‐dimensional electron bolus technique for optimization of postmastectomy irradiation. Int J Radiat Oncol Biol Phys. 2001;51(4):1142–51. 11704339 10.1016/s0360-3016(01)01744-8

[5] Kudchadker RJ , Antolak JA , Morrison WH , Wong PF , Hogstrom KR . Utilization of custom electron bolus in head and neck radiotherapy. J Appl Clin Med Phys. 2003;4(4):321–33. 14604422 10.1120/jacmp.v4i4.2503 PMC5724465

[6] Low DA , Starkschall G , Sherman NE , Bujnowski SW , Ewton JR , Hogstrom KR . Computer‐aided design and fabrication of an electron bolus for treatment of the paraspinal muscles. Int J Radiat Oncol Biol Phys. 1995;33(5):1127–38. 7493839 10.1016/0360-3016(95)00257-X

[7] Zeidan OA , Langen KM , Meeks SL , et al. Evaluation of image‐guidance protocols in the treatment of head and neck cancers. Int J Radiat Oncol Biol Phys. 2007;67(3):670–77. 17197123 10.1016/j.ijrobp.2006.09.040

[8] Zeidan OA , Huddleston AJ , Lee C , et al. A comparison of soft‐tissue implanted markers and bony anatomy alignments for image‐guided treatments of head‐and‐neck cancers. Int J Radiat Oncol Biol Phys. 2010;76(3):767–74. 19427742 10.1016/j.ijrobp.2009.02.060

[9] Low DA , Hogstrom KR . Determination of the relative linear collision stopping power and linear scattering power of electron bolus material. Phys Med Biol. 1994;39(6):1063–68. 15551581 10.1088/0031-9155/39/6/012

[10] Low DA , Starkschall G , Bujnowski SW , Wang LL , Hogstrom KR . Electron bolus design for radiotherapy treatment planning: bolus design algorithms. Med Phys. 1992;19(1):115–24. 1620038 10.1118/1.596885

[11] Bissonnette JP , Moseley DJ , Jaffray DA . A quality assurance program for image quality of cone‐beam CT guidance in radiation therapy. Med Phys. 2008;35(5):1807–15. 18561655 10.1118/1.2900110

[12] Mail N , Moseley DJ , Siewerdsen JH , Jaffray DA . The influence of bowtie filtration on cone‐beam CT image quality. Med Phys. 2009;36(1):22–32. 19235370 10.1118/1.3017470

[13] Yoo S , Yin FF . Dosimetric feasibility of cone‐beam CT‐based treatment planning compared to CT‐based treatment planning. Int J Radiat Oncol Biol Phys. 2006;66(5):1553–61. 17056197 10.1016/j.ijrobp.2006.08.031

